# Complete chloroplast genome of a medicinal species *Polygonatum kingianum* in China (Asparagaceae, Asparagales)

**DOI:** 10.1080/23802359.2020.1721373

**Published:** 2020-01-31

**Authors:** Jian Jin, Jia Lao, Can Zhong, Wei He, Jing Xie, Guoan Hu, Hao Liu, Fulin Yan, Shuihan Zhang

**Affiliations:** a Institute of Chinese Materia Medica, Hunan Academy of Chinese Medicine, Changsha, PR China;; b Hunan University of Chinese Medicine, Changsha, PR China;; c Resgreen Group International Inc, Changsha, PR China;; d Guizhou University of Traditional Chinese Medicine, Guiyang, China

**Keywords:** Polygonatum, high-throughput sequencing, chloroplast, genome sequence

## Abstract

*Polygonatum kingianum* is a medicinal and food plant distributed in most of countries throughout the temperate Northern Hemisphere. Here we report on the complete chloroplast (cp) genome sequence of *P. kingianum*. The cp genome is 155,399 bp in size and includes two inverted repeat regions of 52,7411 bp, which is separated by a large single-copy region of 84,234 bp and a small single copy region of 18,424 bp. A total of 130 genes were predicted, including 38 tRNA, 8 rRNA, and 84 protein-coding genes. Phylogenetic analysis placed *P. kingianum* under the subfamily Nolinoideae of the family Asparagaceae.

The medicinal plant of *Polygonatum* has been traditionally used as tonics in China, and demonstrated to be highly effective in clinical practice for treating age-related diseases, diabetes, lung diseases, fatigue, feebleness and indigestion (Zhao et al. 2018). A strong antioxidant activity was observed by bionic extraction of the rhizome of *Polygonatum* (Jin et al. 2019) and the *α*-glucosidase inhibitors from *Polygonatum* were purified (Zhou et al. 2015). The rhizome of *P. kingianum*, known as “Huangjing” recorded in Chinese Pharmacopeia, is being commonly used part of the plant in traditional Chinese medicine (Jin et al. 2018). However, the information of chloroplast (cp) genome of this medicinal species is limited.

Then, we attempted to characterize the complete cp genome sequence of *P. kingianum* to serve as a valuable genomic resource. Total genomic DNA was extracted from fresh leaves of *P. kingianum* planted in Botanical Garden, Institute of Chinese Materia Medica, Hunan Academy of Chinese Medicine (N28°13′28.15″, E112°56′26.96″). Additional leaf specimens were kept in Hunan Herbarium of Chinese Traditional Medicine under the collection number HUTM100003.

A genomic library consisting of an insert size of 350 bp was constructed using TruSeq DNA Sample Prep Kit (Illumina, USA) and sequencing was carried out on an Illumina NovaSeq platform. The output was a 6 Gb raw data of 150 bp paired-end reads, further trimmed and assembled using SPAdes (Bankevich et al. 2012). Annotations of cp genome were conducted by the software Geneious (Kearse et al. 2012) and further manually checked by comparison against the *P. verticillatum* complete cp genome (GenBank accession number: KT722981).

The complete cp genome of *P. kingianum* (GenBank accession number: MN934979) is 155,399 bp in length, displaying a quadripartite structure that contains a pair of inverted repeats (IR) regions (52,741 bp), separated by a large single-copy (LSC) region (84,234 bp) and a small single-copy (SSC) region (18,424 bp). There are 130 genes reported, including 8 ribosomal RNA genes, 38 rRNA genes, and 84 protein-coding genes. The overall GC content of the cp genome was 37.66%.

To confirm the phylogenetic position of *P. kingianum,* a maximum-likelihood (ML) tree was constructed with 1000 bootstrap replicates using FastTree software (Liu et al. 2011). A subset of another 13 species from the family Asparagaceae was included, with 10 species from Liliaceae as outgroup. The ML analysis showed that *P. kingianum* is placed under the family Asparagaceae, clustered together with other *Polygonatum* species (Figure 1). The taxonomic status of *P. kingianum* exhibits a closest relationship with *P. sibiricum*, *P. verticillatum* and *P. stenophyllum*. This finding could serve as valuable genomic resources providing insight into conservation and exploitation efforts for this important plant species.

**Figure 1.**
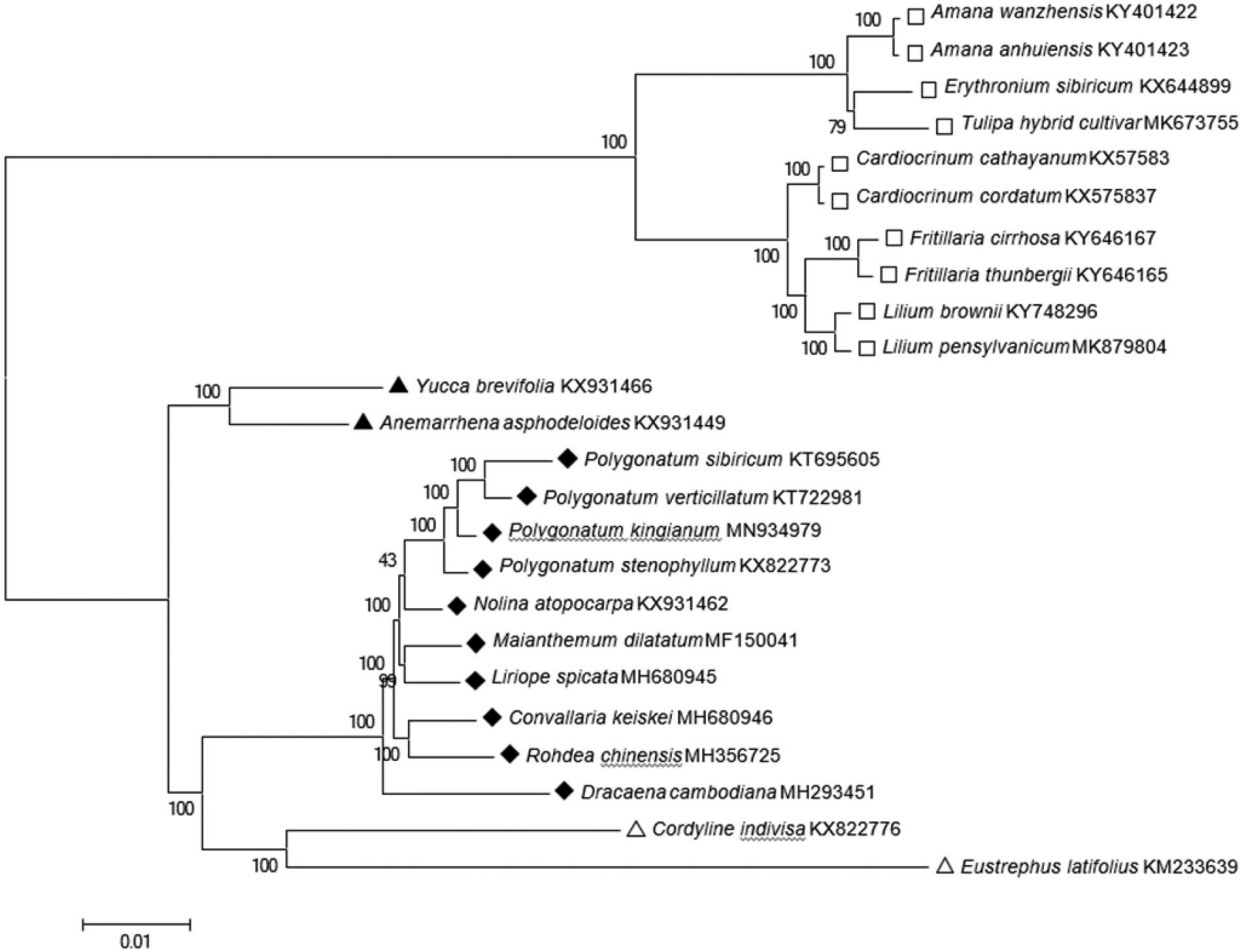
Maximum-likelihood tree based on the complete chloroplast genome sequences of 13 species from the family Asparagaceae with Liliaceae as outgroup. The bootstrap values were based on 1000 replicates.
